# Peer Review of “Utility of the ROX Index in Predicting Intubation for Patients With COVID-19–Related Hypoxemic Respiratory Failure Receiving High-Flow Nasal Therapy: Retrospective Cohort Study”

**DOI:** 10.2196/31895

**Published:** 2021-08-27

**Authors:** Archisman Roy

**Affiliations:** 1 Department of Physics, Faculty of Mathematical Science Institute of Sciences Banaras Hindu University Varanasi India

*This is a peer-review report submitted for the paper “Utility of the ROX Index in Predicting Intubation for Patients With COVID-19–Related Hypoxemic Respiratory Failure Receiving High-Flow Nasal Therapy: Retrospective Cohort Study”.*

## Round 1 Review

### General Comments

The paper [1] is a well-structured piece of research. The authors divided its content into several parts quite brilliantly. It is beneficial and ancillary to the reviewers for easy understanding and commenting. Coming to the subject matter of it, I felt that the content is extensively deep-rooted. The range and the influential spectrum of the paper are indeed broadly scripted. The distinct segmentation of each author’s contribution adds to its vision. To specify the research question beyond drafting the entire write-up and adhering to the focused subject is commendable. The English in use is not so enriched, although the effortless and candid writing makes it suitable for an international journal. In brief, the article is a potentially demanding one. Only a few points can be brought to light for its amelioration. Follow the comments listed below. I am dividing the feedback into major and minor comments. It is requested that you prioritize them.

#### Major Comments

1. Please compose the Objectives subsection under the Abstract differently from the research question. The issue is the same but write that portion in a distinguishable manner.
2. Please discuss “reintubation” and “extubation” separately under the Introduction section. It is the main requirement for the paper.
3. The Methods section seems to be the weakest part of the paper. Please try rewriting this section. I do not feel attaching any information on “who has approved what” is unimportant (within Methods). Please describe the issue of design. If the “design” pertains to methods, the setup, laboratory requirements, or anything else, mention it.
4. What is your unique contribution to respiratory treatment? I am unable to figure it out.
5. To improve readability, the paper should emphasize:
  - Features
  - Models in use
  - Specific methods (which is already in use but the outputs are dynamic)
  - Tabular forms of data sets
  - Relevant outcomes and accuracy
  - Uncertainty and biasedness
6. Induce a section on the limitations and strengths of the article.
7. Please discuss the implication of the application.
8. The text mentions 6 figures, but I cannot find any of them. Please be careful during submission.
9. The authors have discussed the statistical methods in detail, but there is no mention of their application. Please enclose a good deal of statistical analysis.
10. The description is a bit rigorous and difficult to follow. Use some tabular representations of the data. This will allow for less time-consuming and more effective interpretations of the outcomes and results. I am not talking about the data set, but I am emphasizing the outcomes.
11. The Results, Outcome, and Conclusion sections have been written quite well. Please try to improve the way they are presented though. The mathematical sets are lucid enough to understand the results and their nature. However, there is no derivation or any supportive academic background. It is contradicting to the viability as well as the originality of the paper. So, please ensure you have input all the derivations in the text.
12. The citations mentioned throughout the text are indeed following the literature, so the authors’ choice of citations is academically sound.
13. The tables are simple and easy to understand, which is apprehensible.

#### Minor Comments

1. There are too many typos and grammatical issues.
2. Improve the modeling structure of the entire article.
3. Please conform to the authors’ guidelines issued by the publisher.
4. It is expected to have images cited throughout, but the entire text lacks this. Please insert them within the article since it becomes strenuous to follow them otherwise.
5. Please upload supporting data sets in the supplementary materials section.
6. I do not understand what distinguishes “demographics” and the “results” appearing before it.

## Round 2 Review

### General Comments

There is nothing further to comment on the paper. It has been redrafted with a good deal of care. Every bit of it is clearly illustrated. The title is too descriptive but fine. The abstract is clear enough and understandable. Flow charts, figures, and tables have been intriguingly formatted. I enjoyed reading the paper. As mentioned earlier, the article bears acclaimed content along with suitable citations. The writing style and the English in use are adequate.
